# Early Clinical Outcomes of a Simplified Guided Single-Implant Workflow for Mandibular Overdentures: A Prospective Translational Proof-of-Concept Study

**DOI:** 10.3390/dj14100654

**Published:** 2026-10-09

**Authors:** Marco Tudts, Tashia Moodley, Rani D’haese, Stefan Vandeweghe

**Affiliations:** 1 Department of Periodontology, Dental School, Faculty of Medicine and Health Sciences, Ghent University, 9000 Ghent, Belgium; 2 Cosmident, Private Practice, 2500 Lier, Belgium; moodleytashia@gmail.com; 3 Department of Reconstructive Dentistry, Dental School, Faculty of Medicine and Health Sciences, Ghent University, 9000 Ghent, Belgium; rani.dhaese@ugent.be (R.D.); stefan.vandeweghe@ugent.be (S.V.)

**Keywords:** clinical proof-of-concept, edentulous mandible, guided implant surgery, implant-supported overdenture, mandibular overdenture, simplified workflow, single-implant overdenture, translational dentistry

## Abstract

**Background/Objectives:** The present study evaluated the early clinical outcomes and translational proof-of-concept of a simplified guided single-implant workflow for mandibular overdenture rehabilitation in edentulous patients. **Methods:** A prospective translational clinical pilot study was conducted in 15 edentulous patients treated using a simplified flapless guided single-implant mandibular overdenture protocol. The workflow was designed to reduce procedural complexity while maintaining clinically applicable surgical and prosthetic integration under routine treatment conditions. All surgical procedures were performed by a single experienced operator. The study was conducted following institutional ethical approval and informed consent procedures. Clinical, radiographic, peri-implant, prosthetic, and patient-reported outcomes were evaluated during the 1-year follow-up period. Marginal bone loss, peri-implant clinical parameters, complications, implant survival, and Oral Health Impact Profile (OHIP-14) scores were assessed. **Results:** All planned surgical and prosthetic procedures were completed successfully, with no major intraoperative or protocol-related complications. Implant survival was 100% during the 1-year follow-up period. Mean marginal bone loss was 0.19 ± 0.21 mm at loading and 0.25 ± 0.28 mm at 1 year. Peri-implant clinical parameters remained favorable throughout follow-up. Significant improvement in patient-reported quality-of-life outcomes was observed, with OHIP-14 scores demonstrating substantial reduction following treatment (*p* < 0.001). The simplified guided approach demonstrated favorable translational feasibility and practical clinical integration under routine treatment conditions. **Conclusions:** Within the limitations of this prospective translational clinical proof-of-concept study, the proposed simplified guided single-implant workflow demonstrated encouraging early clinical, radiographic, and patient-centered outcomes for mandibular overdenture rehabilitation. Further multicenter investigations involving larger patient cohorts, multiple operators, longer follow-up periods, and comparative study designs are required to evaluate long-term clinical performance, reproducibility, and comparative effectiveness relative to established treatment protocols.

## 1. Introduction

Edentulism remains a significant clinical condition associated with impaired mastication, reduced oral function, diminished prosthesis stability, and decreased quality of life, particularly in elderly populations [1,2]. The aging global population continues to increase the clinical burden associated with edentulism in both developed and developing healthcare settings. Conventional mandibular complete dentures frequently demonstrate limited retention and instability because of progressive alveolar ridge resorption and unfavorable biomechanical conditions within the edentulous mandible [3]. Implant-supported mandibular overdentures have therefore become an important therapeutic approach for improving prosthetic stability, patient comfort, masticatory efficiency, and functional rehabilitation [1,4].

Two-implant mandibular overdentures are widely regarded as a predictable and effective treatment modality and are commonly considered the reference standard for mandibular implant overdenture rehabilitation [1,2]. Nevertheless, despite favorable long-term outcomes, broader clinical implementation of two-implant protocols may remain limited in certain patient populations because of surgical complexity, treatment cost, anatomical limitations, maintenance requirements, and accessibility constraints [5,6]. These considerations have generated increasing interest in simplified implant overdenture approaches capable of reducing treatment burden while maintaining clinically acceptable rehabilitation outcomes.

Single-implant mandibular overdenture concepts have consequently emerged as a potentially simplified treatment strategy for selected edentulous patients [5,7]. Previous investigations have reported encouraging clinical and patient-centered outcomes associated with single-midline-implant overdenture protocols, including improvements in prosthesis retention, oral function, patient satisfaction, and oral health-related quality of life [6,8]. More recently, a systematic review comparing single-midline-implant overdentures with conventional two-implant overdenture systems synthesized evidence from randomized and prospective clinical investigations involving 517 participants and demonstrated that single-implant overdentures may provide clinically acceptable outcomes in selected patient populations while reducing surgical burden, operative time, and procedural cost [9]. Across the included studies, implant survival, crestal bone stability, patient satisfaction, and oral health-related quality-of-life outcomes were generally comparable between treatment approaches, although the available evidence remained heterogeneous and limited by variability in study design, attachment systems, loading protocols, and relatively short follow-up periods. Importantly, Tudts et al. [9,10] emphasized the potential translational value of simplified single-implant overdenture systems in elderly, medically compromised, and economically constrained patient populations where minimizing treatment burden and procedural invasiveness may be clinically advantageous.

In parallel with the evolving clinical evidence base, the integration of digitally guided and flapless surgical workflows has expanded within implant dentistry as a means of improving procedural standardization, surgical precision, reproducibility, and minimally invasive treatment delivery [11,12]. Guided flapless implant surgery may additionally reduce surgical morbidity, operative time, and patient discomfort while preserving periosteal blood supply [13]. A recently published technical proof-of-concept investigation evaluating the guided workflow underlying the present study demonstrated clinically acceptable geometric accuracy and reproducible implant placement across operators with differing levels of implant experience [10]. In that investigation, guided implant placement demonstrated mean deviations of approximately 0.87 mm coronally, 0.98 mm apically, and 6.05 degrees angularly, with no major differences in positional accuracy across operator experience levels. The workflow additionally demonstrated reproducible performance even among less experienced clinicians, suggesting that the guided system may reduce operator-dependent variability during implant positioning. However, the investigation by Tudts et al. [10] primarily focused on technical and imaging-based validation under controlled experimental conditions and did not evaluate translational clinical implementation or patient-centered outcomes under routine treatment conditions.

Taken together, the existing literature therefore establishes a progressive translational foundation for the present investigation. Initial technical validation studies by Tudts et al. [10] demonstrated the geometric feasibility and reproducibility of the guided workflow, while the subsequent systematic review by Tudts et al. [9] supported the potential clinical applicability of single-midline-implant overdenture systems as simplified treatment approaches in selected patient populations. Nevertheless, the translational clinical implementation of simplified guided single-implant workflows remains incompletely characterized. In particular, limited evidence is available regarding practical workflow integration, peri-implant clinical outcomes, radiographic stability, patient-reported outcomes, and overall clinical applicability under routine treatment conditions. Furthermore, early translational implementation studies may face ethical and procedural constraints that differ from those encountered in controlled technical validation investigations, including limitations related to radiation exposure, operator standardization, and workflow integration during routine clinical care.

The present study therefore aimed to evaluate the early clinical outcomes and feasibility of clinical implementation of a simplified flapless guided single-implant workflow for mandibular overdenture rehabilitation. Clinical, radiographic, peri-implant, prosthetic, and patient-reported outcomes were prospectively evaluated during follow-up in order to assess the feasibility and practical clinical applicability of the proposed workflow under routine treatment conditions. The objective of the study was not to establish comparative superiority relative to established two-implant protocols, but rather to evaluate whether the proposed simplified workflow could be predictably implemented as an accessible and clinically practical treatment approach.

## 2. Methods

### 2.1. Study Design

The present investigation was designed as a prospective translational clinical proof-of-concept study evaluating the early clinical outcomes and implementation feasibility of a simplified single-implant guided workflow for mandibular overdenture rehabilitation in edentulous patients. The investigation was conducted as a single-center pilot translational implementation study. The study focused on early-stage clinical translation and practical workflow integration under routine treatment conditions rather than comparative efficacy assessment relative to established two-implant protocols. The study protocol was approved by the Ethics Committee of the University of Ghent, Belgium (Belgian registration number: B670201940410), and performed in accordance with the Declaration of Helsinki and Good Clinical Practice guidelines. The study was designed as a preliminary prospective clinical proof-of-concept investigation. A sample size of 15 participants was considered appropriate for this exploratory evaluation of clinical workflow implementation and early clinical outcomes. Given the limited cohort size, the study was not intended to provide definitive estimates of comparative treatment effectiveness, and the statistical findings should be interpreted within the exploratory nature of the study.

### 2.2. Patient Selection

Fifteen edentulous patients requiring mandibular overdenture rehabilitation were recruited at Ghent University Hospital between October 2019 and December 2023 according to the predefined study protocol. At the initial screening appointment, patients underwent clinical examination and CBCT assessment to determine eligibility. Adequate mandibular symphyseal bone volume for placement of a single midline implant was confirmed radiographically, while clinical assessment was used to determine systemic suitability for implant surgery, oral hygiene compliance, absence of severe parafunctional habits, and suitability for mandibular overdenture rehabilitation. All participants were informed about the nature of the treatment, surgical procedures, potential risks, and follow-up requirements, and written informed consent was obtained before treatment.

Patients with systemic or local contraindications to implant surgery, previous radiotherapy affecting the relevant region, uncontrolled diabetes, active oral infection, severe parafunctional habits, inadequate oral hygiene or inability to comply with oral hygiene and follow-up requirements were excluded. Patients considered clinically or anatomically unsuitable for single-implant mandibular overdenture rehabilitation were also excluded.

All 15 enrolled patients proceeded through the planned treatment protocol and completed the 1-year follow-up. Complete clinical, radiographic, and OHIP-14 data were available for all participants at the relevant assessment points.

### 2.3. Preoperative Assessment and Digital Planning

After enrolment, each patient received a new well-adapted complete denture, which served as the basis for the guided workflow. The removable prosthesis (Merz Dental GmbH, Lütjenburg, Germany) incorporated six 2-mm fiducial glass markers and a polyether ether ketone (PEEK) rail positioned in the mid-symphyseal region. Digital planning was performed using a double-scan CBCT protocol, with the fiducial markers serving as reference points for registration of the datasets. The datasets were evaluated and registered using SIMPLANT (Dentsply Implants NV, Hasselt, Belgium) and ROMEXIS (Planmeca Oy, Helsinki, Finland) software.

The PEEK rail accommodated a movable guide sleeve, allowing adjustment of the planned implant position along the rail. Sleeve angulations of 0°, 12°, or 24° could be selected according to the prosthetically and anatomically appropriate implant trajectory. Guide seating and stability were verified clinically on the basis of the accurate fit and stability of the removable prosthesis. The detailed design and technical principles of the PEEK rail and adjustable guide-sleeve system have been described previously in the proof-of-concept protocol by Tudts et al. [10].

### 2.4. Surgical Procedure

**Figure 1:**
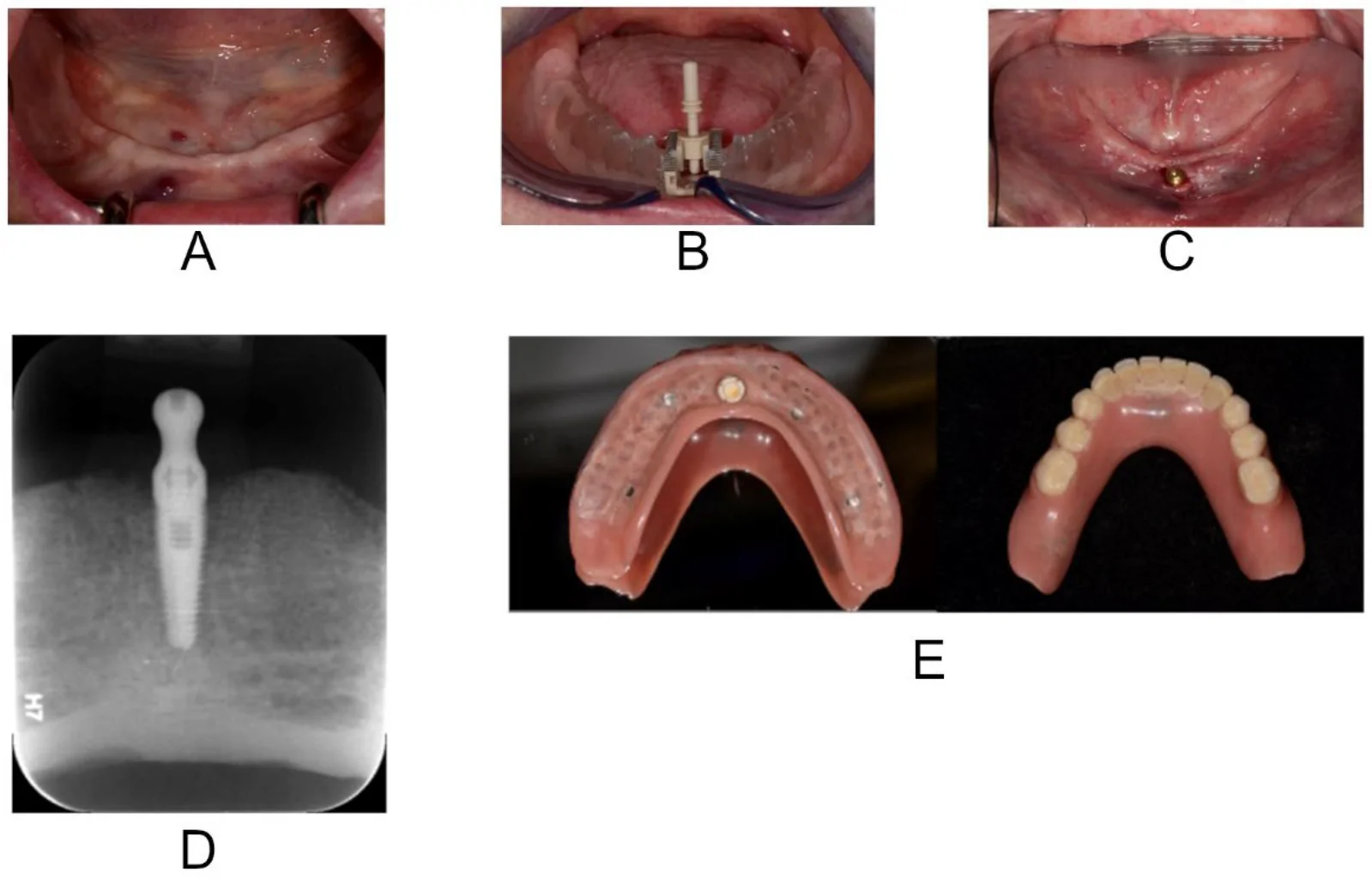
Clinical workflow of single-implant-supported mandibular overdenture treatment and depiction of the surgical protocol employed in the study. (**A**) Preoperative view of the edentulous mandibular ridge. (**B**) Surgical guide positioned in situ during implant placement. (**C**) OBZ abutment secured onto the implant at baseline. (**D**) Baseline periapical radiograph demonstrating implant positioning and peri-implant bone levels. (**E**) Definitive mandibular overdenture prosthesis.

A minimally invasive flapless surgical procedure (Figure 1) was performed to place a single implant in the mid-symphyseal region of the mandible. All implants had a diameter of 4 mm, a tapered body, a hybrid surface configuration, and an external hex connection (Southern Implants Pty Ltd., Pretoria, South Africa). The apical portion featured a moderately roughened grit-blasted surface to promote bone apposition, while the coronal 3-mm region and collar were machined to reduce bacterial adhesion and the risk of peri-implantitis. Before intraoral use, the surgical guide was disinfected using a 0.12% chlorhexidine solution. The removable prosthesis incorporating the PEEK rail and guide sleeve was used as the surgical guide for implant placement according to the CBCT-based plan (Figure 1B). To verify implant placement and ensure accurate and reproducible assessment, a baseline periapical radiograph was acquired using the paralleling technique with a Rinn positioning system (Dentsply Rinn, Elgin, IL, USA). Representative clinical and radiographic stages of the workflow are illustrated in Figure 1A–D. Postoperative care included twice-daily rinsing with 0.12% chlorhexidine digluconate mouthwash for one week and administration of 600 mg ibuprofen or 1 g paracetamol for analgesia.

### 2.5. Prosthetic Procedure

After 3 months, implant osseointegration was assessed using a 20-Ncm reverse torque test. Baseline peri-implant probing depths were measured circumferentially at six standardized, equidistant sites (buccal, lingual, and four interproximal-equivalent sites) around the midline implant. A prototype ball attachment overdenture abutment with increased gingival height (OBZ; Southern Implants Pty Ltd.) was connected to the implant. A periapical radiograph was obtained using the long-cone paralleling technique to confirm proper abutment seating and establish bone levels. Patients were subsequently referred to the prosthetic department for fabrication of the definitive prosthesis. Definitive impressions were taken using a closed-tray technique with copings placed on the seated OBZ abutments, using polyether impression material (Impregum, Solventum, Eagan, MN, USA). A new conventional mandibular denture was fabricated, incorporating a PEEK matrix and a metal intraframe around the OBZ abutment to enhance retention and structural strength (Figure 1E). The definitive prosthetic configuration is illustrated in Figure 1E. Participants received standardized oral hygiene and maintenance instructions. Participants were rehabilitated with a mandibular single-implant-supported overdenture opposing one of the following maxillary conditions: a conventional complete denture (fully edentulous), natural dentition, or a fixed implant-supported prosthesis.

## 3. Clinical and Radiographic Assessment

Participants returned for clinical follow-up 1 year after delivery of the definitive prosthesis. The radiographic assessment comprised three predefined time points: implant placement, loading at 3 months, and 1 year after loading. The radiograph obtained at implant placement served as the initial reference for calculating marginal bone loss at loading, while the 3-month loading radiograph served as the reference for assessing subsequent bone-level change during the 1-year functional loading period. Standardized periapical radiographs were obtained using the paralleling technique and calibrated in ImageJ (version1.54g; National Institutes of Health, Bethesda, MD, USA) using the known implant dimensions. Marginal bone levels were measured mesially and distally from the implant–abutment interface to the most coronal bone-to-implant contact, and the mesial and distal measurements were averaged for analysis. Radiographic measurements were performed independently by two assessors (M.T. and T.M.). Inter-rater reliability was assessed on a subset of 10 radiographs using the intraclass correlation coefficient (ICC), demonstrating good agreement (ICC = 0.85). At the 1-year clinical assessment, probing depths were recorded at six standardized sites around the implant, together with plaque and bleeding assessments.

### 3.1. Outcome Assessment

Clinical, radiographic, peri-implant, prosthetic, and patient-reported outcomes were evaluated throughout treatment and during scheduled postoperative and follow-up visits over the 1-year observation period. The primary outcome measure was marginal bone loss, assessed radiographically at baseline (3 months post-implant placement) and at the 1-year follow-up. Successful workflow implementation was evaluated as a feasibility outcome and was defined as satisfactory guide positioning, completion of the planned guided surgical and prosthetic sequence without conversion to an alternative approach, and absence of intraoperative modifications to the planned workflow. Secondary outcome measures included implant survival, peri-implant clinical parameters (probing depth, modified plaque index, and modified bleeding index where applicable), prosthetic stability and retention, maintenance requirements, oral health-related quality of life, intraoperative and postoperative complications, and the overall practical applicability and successful completion of the simplified guided workflow under routine clinical conditions.

### 3.2. Ethical and Imaging Considerations

Due to ethical considerations related to radiation exposure and adherence to the ALARA (As Low As Reasonably Achievable) principle [14], immediate postoperative CBCT imaging was not performed following implant placement. Consequently, the present investigation was not designed as a geometric accuracy validation study comparable to the earlier technical proof-of-concept investigation by Tudts et al. [10]. Instead, the current study focused on translational clinical implementation, practical workflow integration, and early clinical outcomes under routine patient care conditions.

### 3.3. Oral Health-Related Quality of Life Assessment

Oral health-related quality of life was evaluated using the Dutch version of the Oral Health Impact Profile-14 (OHIP-14) questionnaire [15] before treatment and at the 1-year follow-up. The OHIP-14 comprises 14 items grouped into seven domains: functional limitation, physical pain, psychological discomfort, physical disability, psychological disability, social disability, and handicap. Each item was rated on a 5-point Likert scale, with higher scores indicating poorer oral health-related quality of life. The two corresponding items within each domain were combined to obtain the domain score. For descriptive presentation, an overall mean score across the evaluated OHIP-14 domains was also calculated. Changes in OHIP-14 scores between baseline and the 1-year follow-up were evaluated using paired comparisons.

### 3.4. Statistical Analysis

Descriptive statistics were used to summarize patient characteristics and study outcomes. Continuous variables are reported as mean ± standard deviation or median and interquartile range, as appropriate, and categorical variables as frequencies and percentages. Normality of continuous variables was assessed using the Shapiro–Wilk test. Changes in marginal bone levels between paired assessment time points were analyzed using a paired-samples *t*-test when normally distributed or the Wilcoxon signed-rank test when normality assumptions were not met. Effect estimates are reported with 95% confidence intervals where applicable. Given the exploratory proof-of-concept design and limited sample size (*n* = 15), multivariable regression and subgroup analyses were not used for inferential conclusions. All analyses were performed using IBM SPSS Statistics for Windows, version 22.0 (IBM Corp., Armonk, NY, USA), with statistical significance set at *p* < 0.05.

## 4. Results

**Figure 2:**
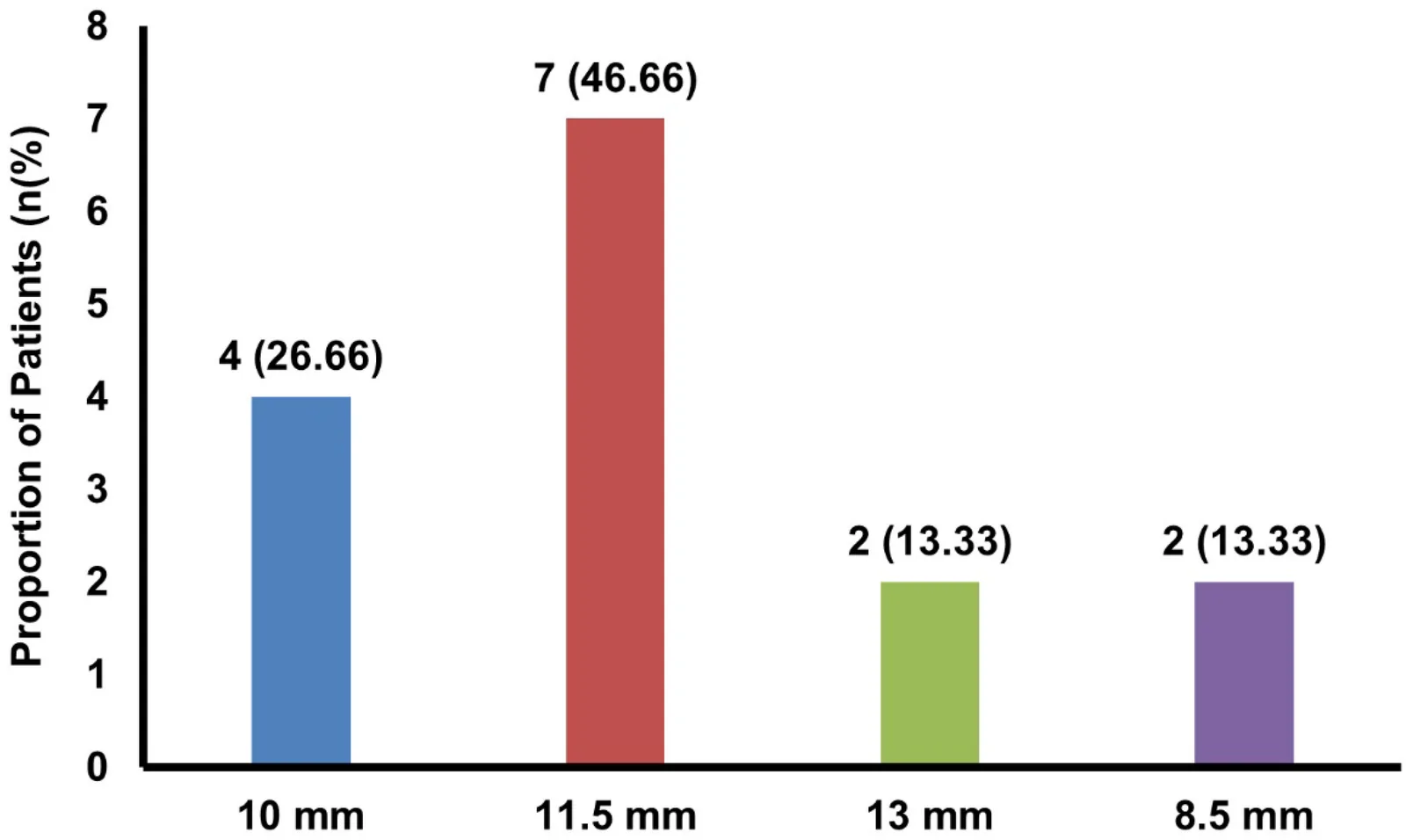
Distribution of implant lengths used in the study cohort.

A total of 15 edentulous patients were enrolled and underwent mandibular rehabilitation using the proposed simplified flapless guided single-implant workflow. All 15 participants completed the planned surgical and prosthetic treatment sequence and the 1-year follow-up, with complete clinical, OHIP-14, and paired radiographic data available for all participants. The cohort included 7 males and 8 females, with a mean age of 60.73 ± 12.18 years (range: 35–81 years). Five participants (33.3%) were smokers. Participants presented with mixed opposing maxillary dentitions, comprising conventional complete dentures, natural dentition, or fixed implant-supported prostheses. Implant lengths were selected according to the available mandibular bone anatomy, with the distribution shown in Figure 2. All implants achieved primary stability exceeding 50 Ncm at placement. All planned surgical and prosthetic procedures were completed without major intraoperative or workflow-related complications. Baseline patient and treatment characteristics are summarized in Table 1.

The guided flapless surgical approach demonstrated favorable clinical integration throughout the treated cohort. Implant placement within the mandibular symphyseal midline region was successfully achieved in all patients using the proposed guided workflow, with completion of the planned surgical sequence and prosthetic integration protocol in all treated cases. Subsequent prosthetic rehabilitation procedures were completed according to the planned treatment protocol, with stable overdenture integration during follow-up.

**Figure 3:**
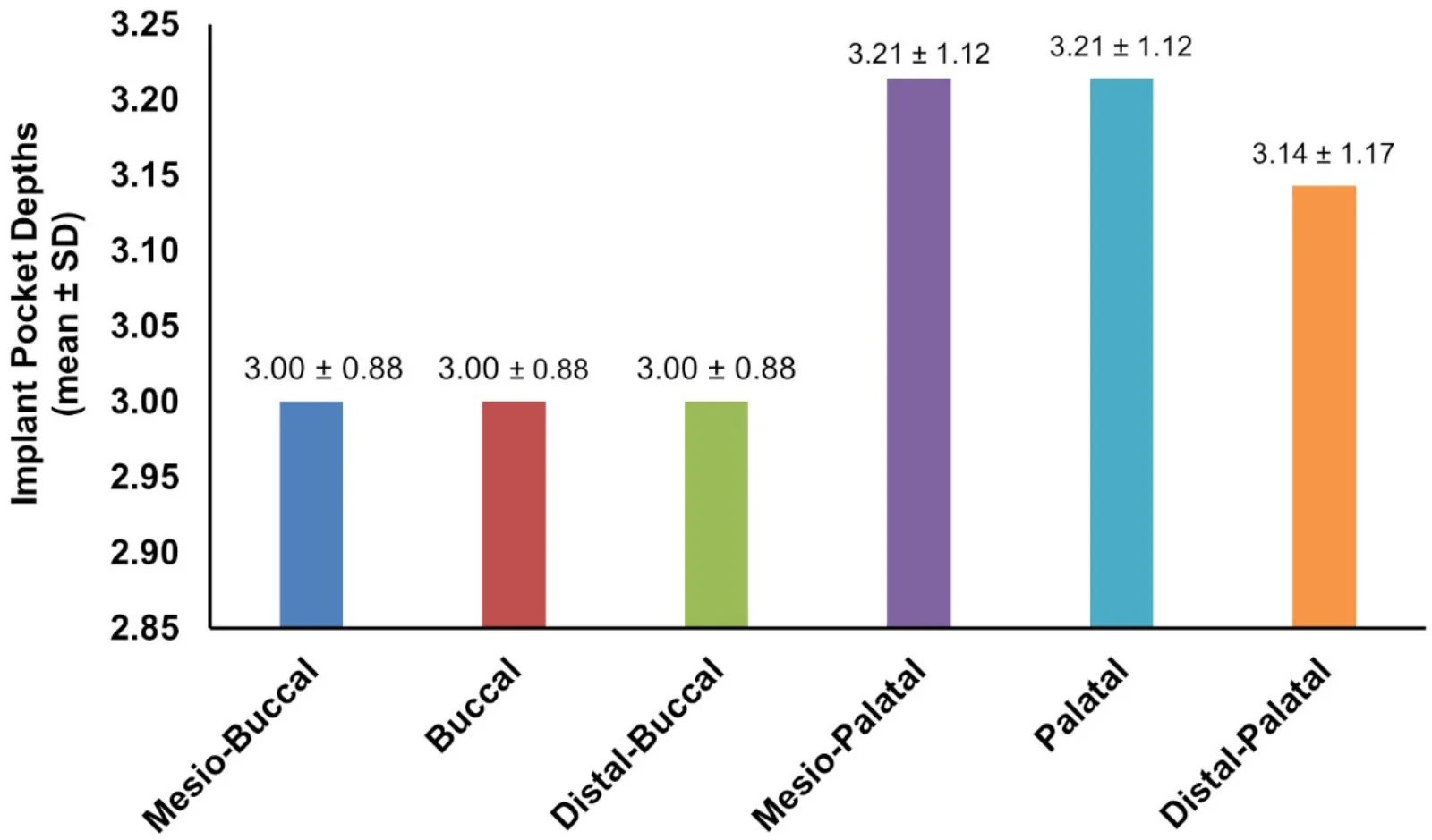
Mean peri-implant probing depths measured at six standardized sites at the 1-year follow-up (*n* = 15).

Implant survival was 100% during the 1-year follow-up period. Peri-implant clinical outcomes were assessed using probing depth, plaque, and bleeding measurements. At the 1-year evaluation, 14 of 15 participants (93.3%) had a plaque index score of 0, while one participant (6.7%) had a score of 1. Peri-implant bleeding was assessed using the Mombelli modified bleeding index, and no bleeding was observed in any participant at the 1-year assessment. Probing-depth measurements at the six standardized peri-implant sites are presented numerically in Figure 3. No major biological or technical complications occurred during follow-up. No prosthetic maintenance events, including matrix replacement, loss of retention, relining, repair, or occlusal adjustment, were required during the 1-year follow-up (Table 2). No major biological or technical complications requiring implant removal, surgical reintervention, or discontinuation of treatment were observed during the evaluation period (Table 2).

Radiographic evaluation demonstrated limited marginal bone loss during follow-up. Mean marginal bone loss was 0.19 ± 0.21 mm at loading and 0.25 ± 0.28 mm at the 1-year follow-up, representing a statistically significant increase over time (95% CI, 0.005–0.100 mm; *p* = 0.018).

**Figure 4:**
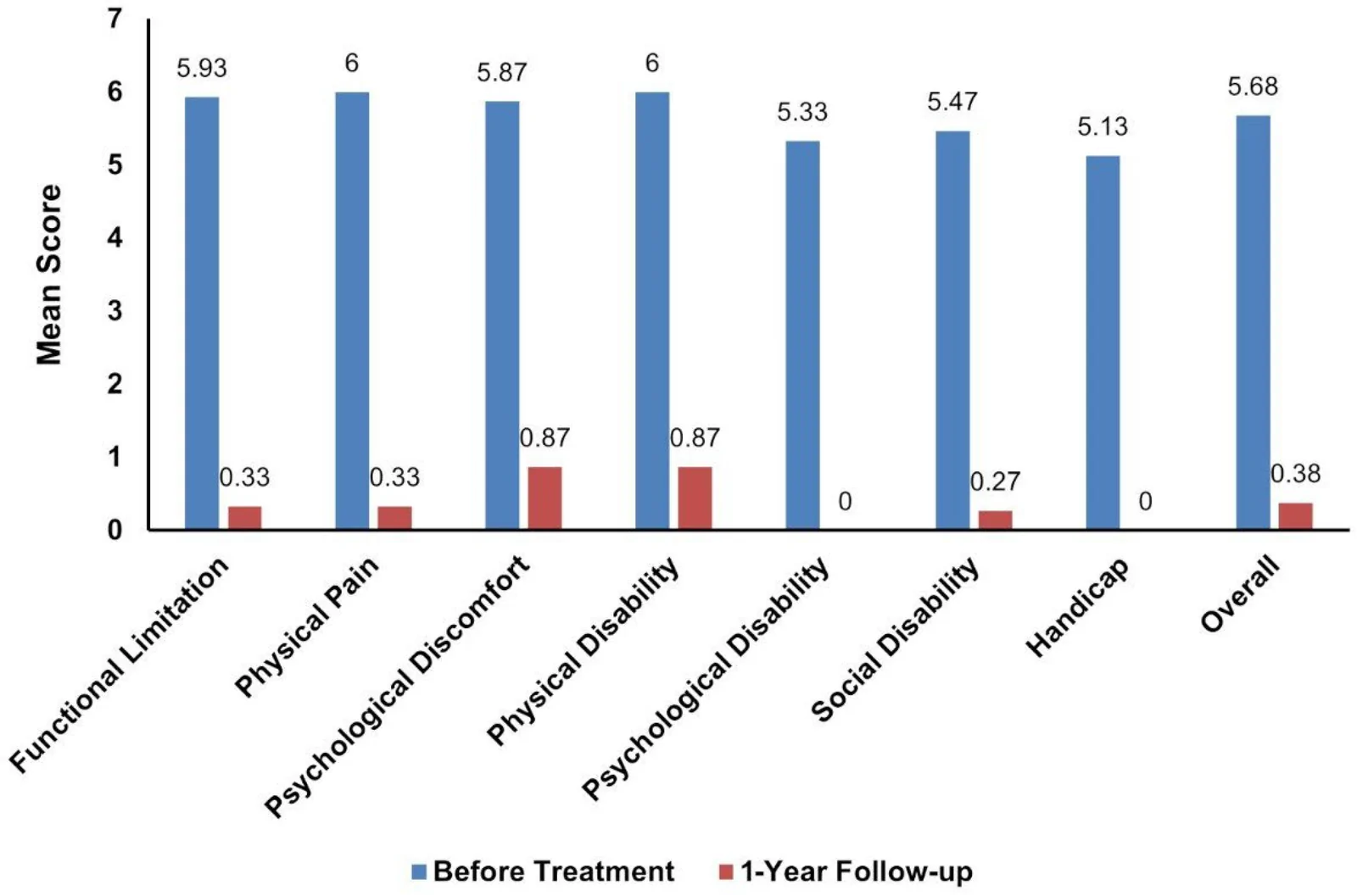
Mean OHIP-14 domain scores at baseline and the 1-year follow-up (*n* = 15). Lower scores indicate better oral health-related quality of life. Values above the bars represent the mean scores.

Patient-reported oral health-related quality of life improved between baseline and the 1-year follow-up. Mean OHIP-14 domain scores decreased from 5.93 to 0.33 for functional limitation, 6.00 to 0.33 for physical pain, 5.87 to 0.87 for psychological discomfort, 6.00 to 0.87 for physical disability, 5.33 to 0.00 for psychological disability, 5.47 to 0.27 for social disability, and 5.13 to 0.00 for handicap. The overall mean score decreased from 5.68 at baseline to 0.38 at the 1-year follow-up (Table 3 and Figure 4).

The planned guided surgical and prosthetic workflow was successfully completed in all 15 patients without conversion to an alternative approach or intraoperative modification. No protocol deviations or major workflow-related complications were observed. These findings support the feasibility of clinical implementation of the workflow within the conditions of this proof-of-concept study.

## 5. Discussion

The present study evaluated the early clinical implementation and outcomes of a simplified flapless guided single-implant workflow for mandibular overdenture rehabilitation in edentulous patients. The planned surgical and prosthetic workflow was completed in all treated patients without conversion to an alternative approach or major workflow-related complications. Implant survival was 100% at the 1-year follow-up, with limited marginal bone loss and satisfactory peri-implant clinical findings. Oral health-related quality of life also improved following treatment, with reductions observed across all OHIP-14 domains (Figure 4). These findings support successful short-term clinical implementation and early clinical performance of the workflow within the conditions of this proof-of-concept study but should not be interpreted as evidence of comparative treatment effectiveness. Mandibular implant overdenture rehabilitation remains an important therapeutic strategy for improving prosthesis stability, masticatory performance, oral function, and patient satisfaction in edentulous populations [1,2,4]. Although two-implant overdenture protocols are widely regarded as a predictable treatment modality and are frequently considered the reference standard for mandibular overdenture rehabilitation [1,16], broader implementation may still be influenced by procedural complexity, treatment cost, anatomical limitations, maintenance requirements, and accessibility considerations in selected patient populations [5,6,7]. Within this context, simplified implant overdenture approaches may provide clinically relevant translational value by reducing procedural burden while maintaining practical rehabilitative utility.

Previous investigations evaluating single-midline-implant overdenture concepts have reported encouraging clinical outcomes and patient-centered improvements in prosthesis retention, oral function, and oral health-related quality of life [5,6,7,8]. More recently, the systematic review by Tudts et al. [9] suggested that single-midline-implant overdenture systems may provide clinically acceptable outcomes comparable to conventional two-implant overdenture approaches in selected patient populations while reducing surgical burden and procedural cost. The present findings are generally consistent with these earlier observations and further support the potential clinical applicability of simplified single-implant overdenture approaches in selected edentulous patients. The favorable marginal bone stability and peri-implant clinical outcomes observed during follow-up additionally suggest that the proposed workflow may provide biologically acceptable short-term clinical performance under routine patient care conditions. The limited marginal bone loss observed during follow-up additionally remained within ranges commonly reported in previous single-implant overdenture investigations.

The rounded design of the OBZ attachment may facilitate peri-implant hygiene and plaque control, potentially contributing to maintenance of peri-implant tissue health. Its design may also influence functional load transfer; however, these potential effects were not specifically evaluated in the present study and require further investigation. In parallel, the increasing integration of digitally guided and flapless implant workflows within prosthodontic rehabilitation has generated interest in treatment approaches capable of improving procedural simplicity, reproducibility, and minimally invasive treatment delivery [11,12]. Flapless guided implant surgery may offer several potential advantages, including reduced surgical morbidity, shorter operative time, improved patient comfort, and preservation of periosteal blood supply [13]. The present study contributes to this evolving translational direction by demonstrating the practical implementation of a simplified guided flapless single-implant workflow within routine clinical care conditions. Importantly, the earlier technical proof-of-concept investigation by Tudts et al. [10] demonstrated acceptable geometric accuracy and reproducible implant positioning across operators with differing levels of implant experience, thereby providing an important technical foundation for subsequent clinical translation of the workflow.

Although several digital implant-prosthetic workflows have been described, including the stackable surgical-guide approach of Yang et al. [17], the Impression Reference technique of Mazzoni et al. [18], and the Smiling Scan and DASS techniques described by Pozzi and co-workers [19,20], these approaches predominantly address immediate-loading full-arch rehabilitation and are therefore not direct clinical comparators for the present single-implant mandibular overdenture protocol. Nevertheless, they share important principles with the present workflow, including prosthetically driven planning, the use of stable reference structures for spatial registration, and guided transfer of the planned implant position to the surgical field. The present protocol differs principally in its simplified application to a single midline mandibular implant. It uses a well-adapted removable prosthesis as the basis of the guide, fiducial markers for dataset registration, and a mid-symphyseal PEEK rail with an adjustable guide sleeve permitting positional adjustment and predefined angulations of 0°, 12°, or 24°. This avoids the more extensive guide assemblies and surgical–prosthetic transfer procedures required for full-arch immediate-loading workflows. The simplification described here therefore relates specifically to the design and number of components required for guided placement of a single midline implant and should not be interpreted as evidence of superiority over established digital workflows.

Collectively, the technical validation study by Tudts et al. [10], the subsequent systematic review by Tudts et al. [9] and the present translational clinical investigation represent sequential stages in the progressive evaluation of the simplified guided single-implant overdenture workflow. Whereas the earlier investigation primarily focused on geometric validation and imaging-based assessment under controlled experimental conditions, the current study was designed to evaluate real-world clinical implementation, peri-implant clinical outcomes, radiographic stability, and practical feasibility within routine patient care conditions. Consequently, the methodological structure of the present study reflected the practical and ethical considerations associated with early-stage clinical translation.

The present investigation was not designed as a comparative efficacy study relative to established two-implant protocols. Rather, it evaluated the short-term clinical implementation of the proposed guided workflow in a selected cohort treated by one experienced operator. The findings should therefore be interpreted within the context of an early translational clinical proof-of-concept investigation and not as evidence of superiority or equivalence relative to established treatment protocols. Furthermore, the observed improvement in OHIP-14 scores reflects the effect of the overall mandibular overdenture rehabilitation and cannot be attributed specifically to the guided surgical component in the absence of a comparator group. One important limitation involved the absence of immediate postoperative CBCT imaging for direct geometric accuracy comparison. This decision was based on ethical considerations related to radiation exposure and adherence to the ALARA principle [14]. As a result, the present study was not intended to function as a formal geometric accuracy validation investigation comparable to the earlier technical proof-of-concept study by Tudts et al. [10]. Instead, the study focused on translational clinical implementation, peri-implant clinical outcomes, radiographic stability, and patient-centered outcomes under routine treatment conditions.

A second limitation relates to the fact that all surgical procedures were performed by a single experienced operator. Although this design may limit generalizability across operators with different experience levels, it also allowed procedural consistency during the early translational implementation phase of the workflow. Similar operator-standardized designs are common within early pilot and translational implant investigations intended to minimize procedural heterogeneity during initial clinical evaluation [21]. Future multicenter investigations involving multiple surgeons and broader patient populations will be important for evaluating reproducibility and operator-dependent variability.

The relatively limited patient cohort should similarly be interpreted within the context of a pilot translational proof-of-concept study. The study was conducted during a period affected by the clinical and logistical constraints associated with the COVID-19 pandemic, which influenced recruitment and reduced the final cohort size relative to the originally planned enrollment. Despite these limitations, the study successfully demonstrated practical implementation of the proposed workflow across the treated cases and generated clinically relevant translational observations that may support future larger-scale investigations.

The findings support further investigation of the guided single-implant workflow for mandibular overdenture rehabilitation. Future studies should include larger patient cohorts, multiple operators, multicenter settings, longer follow-up periods, and comparative study designs to evaluate reproducibility, long-term clinical performance, and comparative effectiveness. More broadly, the present findings support continued progression from technical workflow validation toward broader multicenter clinical evaluation of simplified guided single-implant overdenture systems. Future studies incorporating larger multicenter cohorts, comparative study designs, longer follow-up periods, radiographic evaluation, peri-implant outcome assessment, and patient-reported outcome measures will be required to further evaluate long-term clinical performance, reproducibility, and comparative effectiveness relative to established treatment protocols [1,22,23].

## 6. Conclusions

Within the limitations of this prospective translational clinical proof-of-concept study, the proposed simplified flapless guided single-implant workflow was successfully implemented in all treated patients, with 100% implant survival, limited marginal bone loss, improvement in oral health-related quality of life, and no major workflow-related complications during the 1-year follow-up. In conjunction with the earlier technical validation study and subsequent systematic review by Tudts et al. [9,10], the present investigation represents a further stage in the translational evaluation of the guided single-implant overdenture workflow. The observed clinical and patient-reported outcomes reflect the overall mandibular overdenture rehabilitation and cannot be attributed specifically to the guided surgical component in the absence of a comparator group. Accordingly, these findings support successful short-term clinical implementation in a selected cohort rather than comparative effectiveness relative to established treatment protocols. Further multicenter investigations involving larger patient cohorts, multiple operators, longer follow-up periods, and comparative study designs are required to evaluate reproducibility, long-term clinical performance, and comparative effectiveness.

## Figures and Tables

**Table 1:** Patient and treatment characteristics of the prospective translational clinical proof-of-concept study evaluating a simplified flapless guided single-implant workflow for mandibular overdenture rehabilitation.

Characteristics	Description
Study design	Prospective translational clinical proof-of-concept study
Total patients	15
Patient population	Completely edentulous mandibular patients
Treatment protocol	Simplified single-implant mandibular overdenture workflow
Implant position	Mandibular midline (symphyseal region)
Surgical approach	Guided implant placement
Number of operators	One experienced operator
Prosthetic rehabilitation	Implant-supported mandibular overdenture
Immediate postoperative CBCT	Not performed
Reason for omission of postoperative CBCT	ALARA principle and ethical radiation considerations
Study objective	Translational clinical feasibility and workflow implementation
Major intraoperative complications	None observed
Major workflow-related complications	None observed

**Table 2:** Clinical, Radiographic, and Prosthetic Outcomes During Follow-up.

Outcome Parameter	Result
Implant survival	15/15 (100%)
Marginal bone loss at loading	0.19 ± 0.21 mm
Marginal bone loss at 1 year	0.25 ± 0.28 mm
Paired marginal bone-level change *	0.052 mm
Plaque index at 1 year: score 0	14/15 (93.3%)
Plaque index at 1 year: score 1	1/15 (6.7%)
Bleeding at 1 year	0/15 (0%)
Major biological complications	0
Major technical complications	0
Prosthetic maintenance events	0
Prosthetic failures	0

* Based on all 15 participants with complete paired radiographic measurements.

**Table 3:** Oral Health-Related Quality-of-Life Outcomes at Baseline and 1-Year Follow-up.

OHIP-14 Domain	Baseline Mean	1-Year Mean	Mean Change
Functional limitation	5.93	0.33	−5.60
Physical pain	6.00	0.33	−5.67
Psychological discomfort	5.87	0.87	−5.00
Physical disability	6.00	0.87	−5.13
Psychological disability	5.33	0.00	−5.33
Social disability	5.47	0.27	−5.20
Handicap	5.13	0.00	−5.13
**Overall mean score**	**5.68**	**0.38**	**−5.30**

**Lower OHIP-14 scores indicate better oral health-related quality of life.** Bold is used to adress the overall mean score of all the domains.

## Data Availability

The data presented in this study are available on request from the corresponding author.
